# Droplet-based logic gates simulation of viscoelastic fluids under electric field

**DOI:** 10.1038/s41598-024-52139-8

**Published:** 2024-01-20

**Authors:** F. P Santos, G. Tryggvason, G. G. S. Ferreira

**Affiliations:** 1 Systems Engineering and Computer Science Program, Federal University of Rio de Janeiro, 21941-909 Rio de Janeiro, Brazil; 2 Department of Mechanical Engineering, Johns Hopkins University, Baltimore, MA 21218 USA; 3 Chemical Engineering Program, Federal University of Rio de Janeiro, 21941-972 Rio de Janeiro, Brazil

**Keywords:** Engineering, Mechanical engineering

## Abstract

Nano and microfluidic technologies have shown great promise in the development of controlled drug delivery systems and the creation of microfluidic devices with logic-like functionalities. Here, we focused on investigating a droplet-based logic gate that can be used for automating medical diagnostic assays. This logic gate uses viscoelastic fluids, which are particularly relevant since bio-fluids exhibit viscoelastic properties. The operation of the logic gate is determined by evaluating various parameters, including the Weissenberg number, the Capillary number, and geometric factors. To effectively classify the logic gates operational conditions, we employed a deep learning classification to develop a reduced-order model. This approach accelerates the prediction of operating conditions, eliminating the need for complex simulations. Moreover, the deep learning model allows for the combination of different AND/OR branches, further enhancing the versatility of the logic gate. We also found that non-operating regions, where the logic gate does not function properly, can be transformed into operational regions by applying an external force. By utilizing an electrical induction technique, we demonstrated that the application of an electric field can repel or attract droplets, thereby improving the performance of the logic gate. Overall, our research shows the potential of the droplet-based logic gates in the field of medical diagnostics. The integration of deep learning classification algorithms enables rapid evaluation of operational conditions and facilitates the design of complex logic circuits. Additionally, the introduction of external forces and electrical induction techniques opens up new possibilities for enhancing the functionality and reliability of these logic gates.

## Introduction

Nano and microtechnologies have led to scientific breakthroughs in several disciplines with many applications, such as medicine, biomaterials, solar cells, and energy production^1–4^. Nano and microfluidic technologies are currently acknowledged as emerging tools for preparing drugs with controlled properties and/or creating microfluidic devices similar to logic circuits^5–7^. Thus, there have been significant efforts to elucidate the underlying physics of microfluidic equipment. There is, currently, a particular interest in understanding droplet-based microfluidic devices due to their numerous applications, for example, on-chip separation, biochemical reactor, and logic gates^8–11^.

Exploring viscoelastic droplet logic gates, as discussed by^9^, is part of a quest to discover new ways of computing. This involves using fluid dynamics properties for computation and encoding data beyond traditional electronic parts. Viscoelastic droplet logic can mimic some aspects of biology, which could help identify biological substances, as suggested by^12^. Viscoelastic droplet logic gates could also help create structures using biology-based building blocks and logical operations^13^. There are several applications for droplet-based microfluidic systems with significant scientific impact, especially for those with rigorously controlled dynamics.

Several studies have tried to predict the droplets dynamics in microfluidic devices^14–16^. Most of them are focused on Newtonian fluids^8,17^, although most real applications involve non-Newtonian fluids, such as polymers and emulsions^18^. Sang et al.^19^ investigated the viscosity effect on droplet formation in T-shaped microchannels for three types of continuous fluids; a Newtonian, a non-Newtonian power-law fluid, and a Bingham fluid. They evaluated the effect of the flow behavior index *n*, the coefficient *K*, and $\mathbf {\tau _0}$ (yield stress in the Bingham model), observing that the droplet diameter is strongly dependent on those three parameters. They concluded in their numerical analysis that the droplet size decreases when *K* and *n* increase, and the droplet extension increases with $\mathbf {\tau _0}$. Sontti and Atta compared the micro-droplet dynamics in T-junction^20^ and cross-junction^21^ geometries. They concluded that rheological behavior is a crucial parameter in the shape and size of the droplet formation. Chen et al.^22^ obtained similar results as^19–21^, but stated that the index *n* is more important than *K*.

Qiu et al.^23^ studied the micro-droplet formation numerically and experimentally in a cross-junction for Newtonian and non-Newtonian fluids using the power-law model. They concluded that non-Newtonian liquid flow yields smaller droplet sizes due to its greater shear stress magnitude. Fatehifar et al.^24^ also investigated a non-Newtonian droplet in a Newtonian fluid and the effect on droplet size and different regimes for droplet formation in a cross-junction. Again, the rheological parameters play a critical role in the droplet dynamics and size; however, the authors still use the power-law model. As can be noted, there is a lack of studies for viscoelastic non-Newtonian dispersed phase simulation in the microfluidics literature.

A novel application of droplet-based microfluidics is the logic gate platform. These devices are usually used for diagnostic assay automatization. Despite many bio-fluids, such as blood, exhibiting non-Newtonian characteristics, most studies have been concerned with Newtonian fluids. Droplet-based microfluidics are relatively cheap and versatile, making them extremely attractive for biomedical systems, such as gene sequencing through synthesis^25^. In droplet-based logic gates, the dynamics of the droplet dictate the logic response of the system. The presence or absence of the droplet in the continuous phase usually represents one or zero, respectively, which should have a repeatable dynamic. Anandan et al.^26^ investigated droplet-based microfluidics logic gates using the phase-field method for four computational geometry models. They analyzed different operating conditions to optimize and parameterize the geometries and the input flows according to defined specifications. However, once again, their working fluids were Newtonian fluids. In the same direction, Yang et al.^6^ examined a logic device based on the particle dynamics in viscoelastic fluid focused on blood flow. They modeled the continuous fluid with the Oldroyd-B model. The particles were Lagrangian for a wide range of operations for $\textbf{XOR}$, $\textbf{OR}$, $\textbf{AND}$, and $\textbf{NOT}$ gates. However, in this Lagrangian approach, the droplets cannot deform.

Asghari et al.^8^ studied droplet-based microfluidics logic gates with a non-Newtonian power-law model and also evaluated a specific $\textbf{AND}$/$\textbf{OR}$ geometry configuration. The authors study the effects of relevant parameters: the power-law index, the droplet length, the capillary number, and the geometrical parameters of this system. They identified the regions where the logic operation would be possible in a sort of regime map. Although it is necessary to study viscoelastic fluids in logic gates microfluidics, as far as the authors’ knowledge, no droplet-based logic gate study considers a viscoelastic model for deformable droplets. Accurate modelling of the viscous stresses in the fluid is essential because the droplet dynamics (breakup, deformation, and droplet generation) strongly depends on the relation between shear stress and the shear strain rate, which plays a vital role for logic gates devices. It is expected that a viscoelastic fluid in this system can operate differently from other non-Newtonian fluids that follow the power-law models.

Electrically driven droplets are an alternative to control the dynamics of microfluidic logic devices. They have been proven to be an interesting tool in different applications because they respond relatively fast and are robust to control^27–29^. Electrostatic forces in microfluidic devices were used experimentally for the first time by^30^. They inferred that droplets react with different levels of deformation depending on the magnitude of the electrical stress. In another direction, Wehking et al.^31^ and Wehking and Kumar^32^ studied the droplet dynamics by numerical simulations under a direct current electric field in a microchannel. In their work, the droplet deformed, squeezed, decelerated, and pinned depending on the magnitude of the electric potential applied. Xi et al.^33^ proposed an experimental analysis of droplet-based microfluidic with electric field in cross-junction geometry. They corroborate that the droplet deformation is strongly dependent on the electric field intensity. Li and Zhang^34^ studied the electro-hydrodynamic of droplet generation in a microfluidic device where the droplet is formed by inducing a polarization in the droplet by the electric field. They concluded that the droplet size varies with the electric capillary number, which means that an electric force induction can control the droplet size in this process. Yin et al.^35^ took the same approach but with a different geometry employing experiments and numerical simulation. They observed that the droplet size becomes smaller when the electric potential increases, and the electric capillary number is the most relevant parameter. While some microfluidics initiatives using electric fields are focused on droplet manipulation^31,32,34,35^; none of them focus on logic gates devices.

In this present work, we provide: a systematic analysis of non-Newtonian viscoelastic fluid with Oldroyd-B model, considering the variation of Weissenberg number (Wi), Capillary number (Ca), and geometric parameters. A non-operating (when the logic gate does not work correctly) region may become operational (when the logic gate works properly) if an external force is applied. We also proposed an electrically induced technique to transform non-operational conditions into operational ones using an alternating current electric field. Instead of producing a map where the operation is feasible, as in^8^, we conclude by providing a classification neural network to identify the regimes where the $\textbf{AND}$/$\textbf{OR}$ logic gate is operational.

## Mathematical models and numerical methods

In order to simulate the complexity of electrohydrodynamic phenomena, a formulation for describing how the electric field affects the droplet dynamics is needed. Here, a charge-conservative equation to solve multiphase electrohydrodynamic problems is developed by employing the volume of fluid method and Maxwell equations as electroquasistatic^36^, ignoring the magnetic effects. For the behavior of the phases, a volume of fluid (SVOF) method is used to capture the interface, and the iso-advector^37^ to reconstruct the interface position and curvature. For the capillary stress and the electric stress, the continuous surface tension force methodology and Maxwell’s equation is coupled with the SVOF and the iso-advector approach since the curvature must be calculated accurately. This section presents the mathematical and numerical models utilized in this work.

### Immiscible two-phase model

The governing equations for multiphase flow are the momentum equations, with the interface forces incorporated as a source term, as in^38^. The total mass conservation equation, where $\rho$ and ${\textbf{u}}$ are the density and velocity field, respectively, is:

1 $$\begin{aligned} \frac{\partial \rho }{\partial t} + \nabla \cdot \left( \rho {\textbf{u}} \right) = 0. \end{aligned}$$

The momentum equations for incompressible, viscous, and immiscible two-fluid systems can be written as:

2 $$\begin{aligned} \rho \left( \frac{\partial {\textbf{u}} }{\partial t} + {\textbf{u}} \cdot \nabla {\textbf{u}} \right) = - \nabla p + \rho {\textbf{g}} + \nabla \cdot \mathbf {\tau } + \mathbf {F_{\gamma }} + \mathbf {F_e}, \end{aligned}$$

where ${\textbf{g}}$ and *p* are gravitational acceleration and pressure, respectively. $\nabla \cdot \mathbf {\tau }$ is the viscous force. $\mathbf {F_e}$ is the electric body force calculated from the Maxwell stress tensor acting at the fluid-fluid interface^39^. $\mathbf {F_{\gamma }}$ is the surface tension force which is modeled as a volumetric force using the continuum surface force (CSF):

3 $$\begin{aligned} \mathbf {F_{\gamma }} = \gamma \kappa \nabla \alpha , \end{aligned}$$

where $\gamma$ is the surface tension, $\alpha$ is the fluid volume fraction and $\kappa$ is the interfacial curvature defined as:

4 $$\begin{aligned} \kappa = \nabla \cdot \left( \frac{\nabla \alpha }{\arrowvert \nabla \alpha \arrowvert } \right) . \end{aligned}$$

The volume of fluid (VOF) approach uses the volume fraction conservation equation to capture the interface,

5 $$\begin{aligned} \frac{\partial \alpha }{\partial t} + \nabla \cdot \left( {\textbf{u}} \alpha \right) = 0. \end{aligned}$$

The multiphase system thermophysical properties are calculated according to:

6 $$\begin{aligned} \theta = \theta _1 \alpha + \theta _2(1- \alpha ), \end{aligned}$$

where $\theta$ is any mixture property, calculated by a weighted average of properties of the pure fluids ($\theta _1$ and $\theta _2$) with respect to their volumetric fractions.

### Viscoelastic model

Here, we are interested in viscoelastic fluids. Thus, to predict the viscous force, $\nabla \cdot \mathbf {\tau }$, we used the Oldroyd-B model^40^. This constitutive model describes a polymeric stress tensor (with large deformations), where an extension of the Upper Convected Maxwell model represents an idealized fluid with elastic bead and spring dumbbells. In Oldroyd-B, the viscoelastic part is separated from the Newtonian part as follows:

7 $$\begin{aligned} \mathbf {\tau } = \mathbf {\tau _s} + \mathbf {\tau _p}, \end{aligned}$$

where $\mathbf {\tau _s} = \mu _s {\textbf{S}}$ is the solvent stress tensor. In this case, ${\displaystyle {\textbf{S}} }$ is the deformation rate tensor or rate of strain tensor, ${\displaystyle {\textbf{S}} ={\frac{1}{2}}\left[ {\varvec{\nabla }}{\textbf{v}} +({\varvec{\nabla }}{\textbf{v}} )^{T}\right] }$, and $\mu _{s}$ is the Newtonian viscosity. $\mathbf {\tau _p}$ is the viscoelastic stress tensor whose behavior is described as:

8 $$\begin{aligned} \mathbf {\tau _p} + \lambda \overset{\bigtriangledown }{\mathbf {\tau _p}} = \mu _p {\textbf{S}}, \end{aligned}$$

where $\mu _{p}$ is the viscoelastic viscosity, ${\displaystyle \lambda }$ is the relaxation time and ${\mathbf {\tau _p}}$ is the upper-convected time derivative of the stress tensor, described by:

9 $$\begin{aligned} \overset{\bigtriangledown }{\mathbf {\tau _p}} \equiv \frac{\partial \mathbf {\tau _p}}{\partial t} + \nabla \cdot \left( {\textbf{u}} \mathbf {\tau _p} \right) - \left( \nabla {\textbf{u}} \right) ^{T} \cdot \mathbf {\tau _p} - \mathbf {\tau _p} \cdot \nabla {\textbf{u}}. \end{aligned}$$

### Maxwell equations

Herein, the Maxwell equations are approximated as electroquasistatic, ignoring the magnetic effects^36,39^. As the dynamic currents are small, the electric field is irrotational; thus, we have:

10 $$\begin{aligned} \nabla \times {\textbf{E}} = 0, \end{aligned}$$

where ${\textbf{E}}$ is the electric field. After applying Gauss’ law, Eq. (10) can be reduced to:

11 $$\begin{aligned} \nabla \cdot \left( \epsilon {\textbf{E}}\right) = \rho _e, \end{aligned}$$

where $\epsilon$ and $\rho _e$ are the dielectric permittivity and the bulk-free charge density, respectively. The conservation equation for the bulk free charge density is based on the assumption that each fluid has different electrical properties, yielding:

12 $$\begin{aligned} \frac{\partial \rho _e}{ \partial t} + \nabla \cdot \left( {\textbf{u}} \rho _e \right) = - \nabla \cdot \left( \sigma {\textbf{E}} \right) , \end{aligned}$$

where $\sigma$ is the conductivity.

Finally, the Maxwell stress, $\mathbf {\tau }^{e}$, neglecting the electrostriction effect, can be expressed as:

13 $$\begin{aligned} \mathbf {\tau }^{e} = \epsilon \left[ {\textbf{E}}{\textbf{E}} - \frac{1}{2}\left( {\textbf{E}}\cdot {\textbf{E}}\right) {\textbf{I}}\right] , \end{aligned}$$

where ${\textbf{I}}$ is the second-order identity tensor. The bulk electric force, present in Eq. (2), can be derived from the divergent of Maxwell stress tensor, Eq. (13):

14 $$\begin{aligned} \mathbf {F_e} = \nabla \cdot \left( \mathbf {\tau }^{e} \right) = \rho _e {\textbf{E}} - \frac{1}{2}E^{2}\nabla \epsilon . \end{aligned}$$

### Numerical approach

The electrohydrodynamic multiphase flow framework was implemented and simulated in OpenFOAM^41^, a C++ open-source project for developing customized numerical solvers specialized in computational fluid dynamics (CFD), due to its large number of solvers and utilities. In order to simulate the droplet dynamics in the logic gate device, we developed a numerical solver based on an open-source toolbox to simulate the flow of viscoelastic fluids called RheoTool^42^, based on OpenFOAM-4.0.

The interIsoFoam solver, a VOF method that tracks the fluid-fluid interface, was adapted to include the RheoTool viscoelastic library. In interIsoFoam the CSF model calculates the surface tension effects, and the iso-advector^37^ algorithm reconstructs the interface position and curvature. To predict the electric force (Eq. 14), the charge-conservative equations (Eqs. 11, 12) were implemented as a library in OpenFOAM^41^. Then, Eq. (14) is included as a source term in Eq. (2). The resulting solver includes three multiphysics models: several viscoelastic fluid models, the electroquasistatic model, and the Two-Phase Immiscible model, based on iso-advector interface reconstruction.

The pressure implicit with splitting of operators (PISO) algorithm is used to couple the pressure-velocity in the momentum equation^43^. The electroquasistatic model (Eqs. 11, 12) is evaluated sequentially after updating the pressure equation in the PISO loop, and the electric force (Eq. 14) is included in the momentum equation explicitly. Then, the effect of changes in charge density and the electric field is fully incorporated in the pressure-velocity coupling algorithm, ensuring a global convergence of the system of equations.

**Figure 1:**
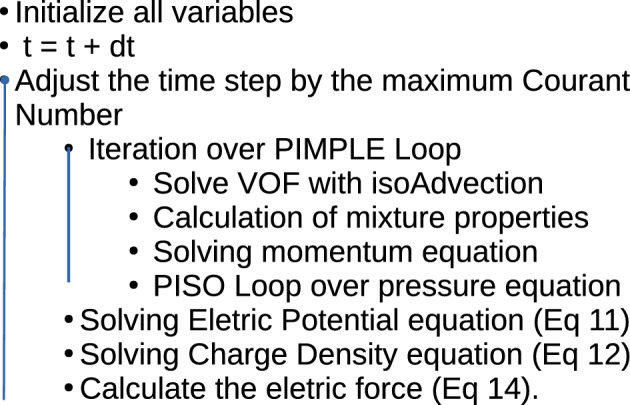
Algorithm proposed for the electrohydrodynamic solver.

The temporal terms are discretized using a second-order implicit Euler scheme. Spatial discretization is performed using a second-order upwind scheme for the momentum equation and van Leer limiter to keep the phase fraction advection bounded. A linear scheme was applied for the Laplacian operator, which is also second order accurate. In order to respect the Courant–Friedrichs–Lewy (CFL) condition, the time step is limited by a fixed Courant Number (*Co*) less than 0.3 for the whole domain and all equations. Gmsh^44^, a free mesh generator, was used to build the geometries and the meshes. See in Fig. 1 the algorithm proposed for simulatons:

### Simulated conditions

In this section, we describe the physical conditions of the simulations used in code verification, in the logic gate evaluation under viscoelastic fluid flow and in the training of the Binary Classification ROM.

To verify the multiphysics electrohydrodynamic solver, a circular droplet suspended in an immiscible fluid is placed between two parallel eletrodes with a constant electric field. We perform simulations for both Newtonian and viscoelastic fluids, and compared the observed deformation against predictions of the Taylor-theory^45^. The distance between the two plates is $H_d = 10R_d$, where $R_d$ is the initial radius of the droplet. Here, three dimensionless parameters are defined, $R =\frac{\sigma _1}{\sigma _2}$, $\beta = \frac{\nu _1}{\nu _2}$ and $Q =\frac{\epsilon _1}{\epsilon _2}$, where $\nu = \frac{\mu }{\rho }$ and the subscript denotes fluid one or two, dispersed and continuous phase, respectively. As in^46^, the simulations were performed in an axisymmetric geometry. The computational domain has $200\times 200$ mesh nodes with $R_d = 0.1$ cm, leading to a domain physical size of 1 cm. At the electrode walls, a no-slip velocity condition with constant electric potential was prescribed. The potential values were specified based on the capillary number, $Ca_E = \frac{ \epsilon _2 R_d {\textbf{E}}\cdot {\textbf{E}} }{\gamma }$.

The Newtonian droplet simulation use conditions similar to those used by^46^, with varying $2< R < 14$ and fixed $Q=10$, $Re = \frac{U R_d}{\nu _2} = 0.09$, $Ca_E = \frac{ \epsilon _2 R_d {\textbf{E}}\cdot {\textbf{E}} }{\gamma } = 0.18$ and $\beta = \frac{\nu _1}{\nu _2} = 1$. According to^45^ theory, the deformation parameter, *D*, can be expressed as a function of the fluid properties and the electric field strength,

15 $$\begin{aligned} D = \frac{9}{16} \frac{Ca_E}{(2 + R)^2} \left[ 1 + R^2 - 2Q + \frac{3}{5}\left( R-Q\right) \frac{2+3\beta }{1+\beta }\right] = \frac{H-L}{L+H}, \end{aligned}$$

where *H* and *L* are the droplet lengths perpendicular and parallel to the plates, respectively. This can be explained by the discretization errors of the three meshes: $100\times 100$, $200\times 200$ and $300\times 300$ mesh nodes. In these cases, the estimated mesh error was between $6\%$ and $8\%$ for *D*, which can cause a significant effect in the interface curvature for cases with static droplets.

In the viscoelastic fluid flow cases, the simulation conditions follow the work of^47^. In this case, the viscoelastic parameter was specified based on the Weissenberg number, $Wi = \frac{U \lambda }{H_d}$, where $\lambda$ is the relaxation time. The droplet deformation was computed for a range of varying Capillary number ($0.1< Ca < 2.5$) with fixed $Wi = 1$, $\lambda = 1$, $R= 2.5$ and $Q = 2.0$, and the results are compared against the predictions of the Taylor-theory and the simulations of^47^.

**Figure 2:**
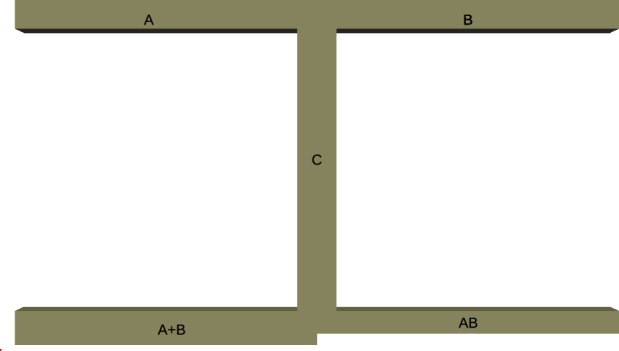
Logic gate geometry. Channels ${\textbf{A}}$ and ${\textbf{B}}$ are inlets; and $\mathbf {A+B}$ and $\mathbf {A\cdot B}$ are outlets.

To evaluate the effect of viscoelastic fluids on a logic gate system, a modified geometry version of the system proposed by^48^ and analyzed by^8,49^ was used as a reference to verify our proposed methodology, as shown in Fig. 2. As in^8,49^, the inlet channels, named Tubes ${\textbf{A}}$ and ${\textbf{B}}$, have width of 50 $\upmu$m and length of 500 $\upmu$m. Tube $\mathbf {A+B}$ has a width of 50 $\mu m$ and a length of 500 $\upmu$m, and Tube $\mathbf {A.B}$ has a width of 25 $\upmu$m and a length of 500 $\upmu$m. Thus, Tube $\mathbf {A+B}$ has less hydrodynamic resistance since it has a larger hydraulic diameter than Tube $\mathbf {A.B}$. It is expected that droplets coming from ${\textbf{A}}$ and/or ${\textbf{B}}$ will flow preferably to branch $\mathbf {A+B}$ due to its lower resistance. However, based on the balance between the hydrodynamic resistance and the resistance created by the blockage of an existing droplet, the droplet can choose between $\mathbf {A+B}$ or $\mathbf {A.B}$. The droplet logic gate definition of $\textbf{AND}$ and $\textbf{OR}$ states takes into account these different dynamics. In our case, **O** **R** logic occurs when one droplet comes from either ${\textbf{A}}$ or ${\textbf{B}}$ and “decides” to go to $\mathbf {A+B}$. $\textbf{AND}$ happens when two droplets coming from ${\textbf{A}}$ and ${\textbf{B}}$ get together in a big droplet in the Tube ${\textbf{C}}$ and eventually is split between tubes $\mathbf {A+B}$ and $\mathbf {A.B}$. If the system respects either $\textbf{AND}$ or $\textbf{OR}$ gate, we classify the system as operational.

The boundary conditions for the simulations are Dirichlet boundary conditions for the velocity inlet, with different values specified depending on the case studied. For the pressure field, the Neumann boundary condition and thus zero gradients are specified. Zero gradients for velocity and constant pressure are set up in the outlet. At walls, the no-slip condition is enforced because, in this case, the slip length scale is of the order of magnitude of a nanometer, and the size of our geometry is in the micrometer size range. The first step is to obtain the best grid size for the simulations. Meshes of 28,918 (mesh 1), 107,888 (mesh 2), and 135,329 (mesh 3) nodes were used in the grid independence analysis. To evaluate the mesh convergence, the viscoelastic phase volume fraction along the tubes $\mathbf {A+B}$ and $\mathbf {A.B}$ in the middle of the tube $\mathbf {A.B}$ was compared for each mesh. In order to evaluate the worse scenario, the convergence is studied for the case with $L/H = 1.4$, $Ca = 0.1$ and $Wi = 4$ for the $\textbf{AND}$ at the time ($t = 1.5\times 10^{-2}$ s) when the droplet reaches the connection between tubes ${\textbf{C}}$, $\mathbf {A+B}$ and $\mathbf {A.B}$.

### Classification model

In binary logic gate applications, one is interested in classifying data between two states. It is straightforward to realize that the binary neural network classification model is the right tool to predict the droplet-based logic gate state. All classification tasks depend upon labelled datasets; in our case, this dataset is provided by numerical simulation that transfers their knowledge to a neural network to learn the correlation between labels and data. Here, in order to build a reduced-order model (ROM) to predict operational conditions, several computational fluid dynamics simulations were performed to generate the dataset for the neural network, whose input data are the relevant physical dimensionless numbers of the problem.

The TensorFlow^50^, open-source software library developed by Google was used for the classification model training. It provides an interface for expressing machine learning algorithms and an application for executing these algorithms. For binary classification, the binary cross-entropy loss’ function and Adam optimization algorithm optimizer were used to build our deep neural network logic gate ROM because it is a robust method to solve non-linear multidimensional classification problems, as in our case. Details related to the parameters of the dense neural network structure presented in this work will be provided in “Results and discussion” section.

We seek for parameter $\Gamma$ that minimizes the negative binomial log-likelihood (Eq 16).

16 $$\begin{aligned} Loss(\Gamma ) = -\frac{1}{N_{train}}\sum _{i = 0}^{N_{train}}\left( y_i\log (G(\mathbf {x_i},\Gamma )) + (1-y_i)\log (G(\mathbf {x_i},\Gamma ))\right) \end{aligned}$$

where $Loss(\Gamma )$ is the loss function to be minimized. The loss function uses $y_i$ and $G(\mathbf {x_i},\Gamma )$ as probability distributions and measures the discrepancy between the neural network and the label, and $\mathbf {x_i}$ are its input vector. The training process happens iteratively by computing loss function, $Loss(\Gamma )$, and updates the model until convergence. The trained model is then tested to assess metrics like accuracy and precision.

## Results and discussion

This section is divided into three parts: in the first part, a code verification is performed for both a Newtonian and a viscoelastic droplets in an electric field, by comparing well-known analytical results for the Newtonian case and numerical results for the non-Newtonian case. In the second part, the influence of the viscoelastic surface and geometric parameters of the logic gate are analyzed. All data generated in the previous step was used to produce a binary classification model. Then, in the third part, we propose an electrically induced droplet formation method to control the logic gates system.

### Verification: droplet deformation under electric field

**Figure 3:**
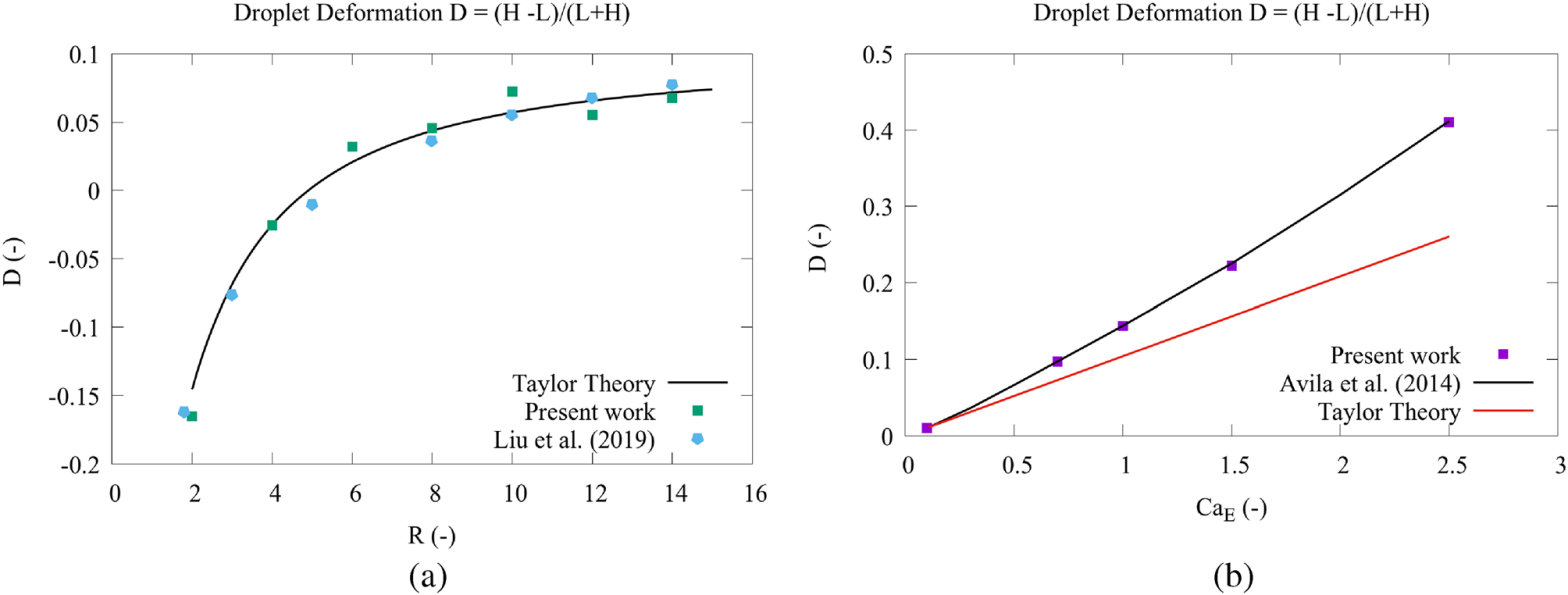
Droplet deformation, *D*, versus the dimensionless parameter *R* for a Newtonian fluid for $Q=10$ for the Newtonian case, specifying the $Re = \frac{U R_d}{\nu _2} = 0.09$, $Ca_E = \frac{ \epsilon _2 R_d {\textbf{E}}\cdot {\textbf{E}} }{\gamma } = 0.18$ and $\beta = \frac{\nu _1}{\nu _2} = 1$. (**a**) Versus the capillary number $Ca_E$ for a viscoelastic fluid for $R= 2.5$ and $Q = 2.0$ (**b**).

In this section we describe the results obtained for the deformation of a Newtonian and a viscoelastic fluid droplet under a fixed electric field. In Fig. 3a, we show the obtained values for the droplet deformation parameter *D* under different $R = \frac{\sigma _1}{\sigma _2}$ for a Newtonian fluid droplet. One can see that our numerical results agree with the results obtained by^46^ and with the theoretical predictions of^45^ when we compare the droplet deformation *D* over time. There exists a slight discrepancy between the result presented here and^46^. The reason for this discrepancy can be interpreted as a result of spurious current present in the volume of fluid approach. We performed a mesh convergence analysis for this case by considering three mesh sizes ($100\times 100$, $200\times 200$ and $300\times 300$ discretization nodes), and we concluded that the estimated mesh error was between $6\%$ and $8\%$ for *D*, which can cause a significant effect in the interface curvature for cases with static droplets. Figure 3b shows the the droplet deformation parameter *D* under different $Ca_E$ in a viscoelastic fluid droplet. It can be observed that the results presented here agree very well with the simulation result obtained by^47^. However, there is an enormous discrepancy between the Taylor theory linear analysis. This result was expected since all Taylor’s theory is based on a Newtonian fluid and for a low $Ca_E$ number. As was also expected, for a low $Ca_E$ number, the deformation experienced by the viscoelastic fluid droplet is similar to a Newtonian fluid droplet because the electric field effect is too weak to produce an effective viscoelastic response. As the $Ca_E$ increases, the difference between the analytical and numerical solutions increases.

### Microfluidic logic gates

#### Mesh independency analysis

**Figure 4:**
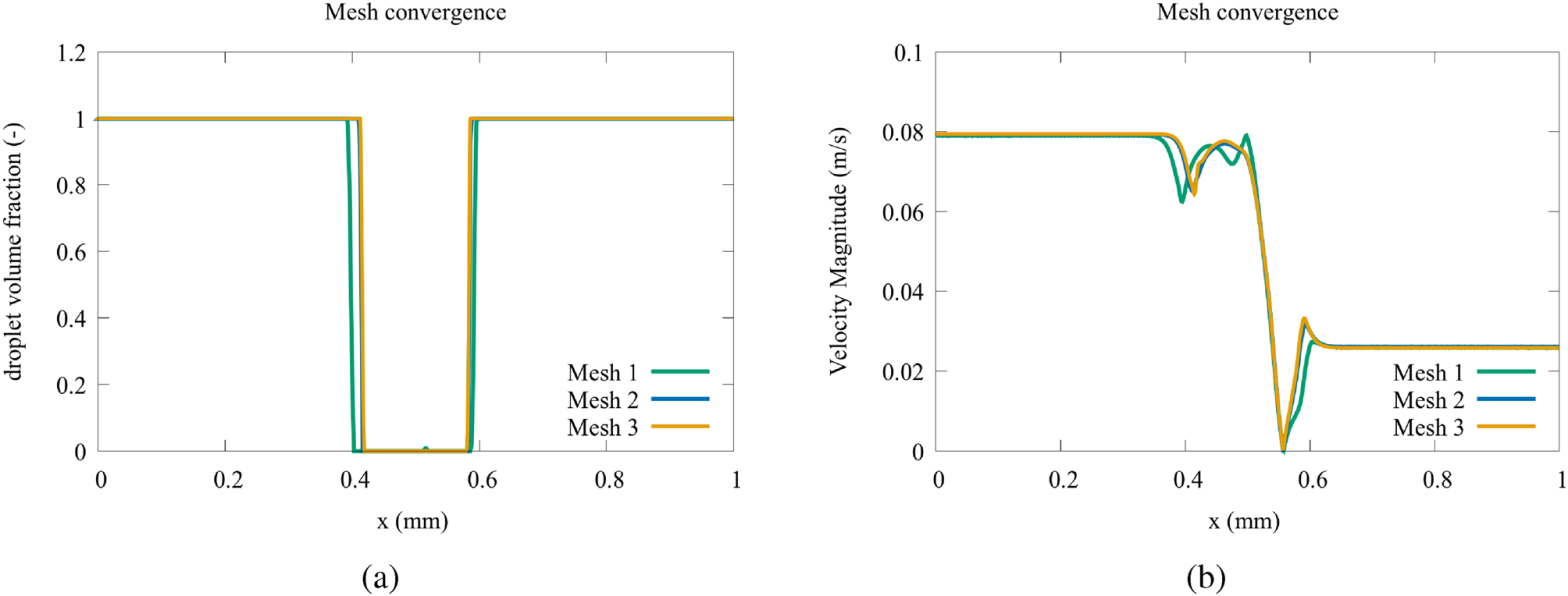
Comparison of velocity magnitude [m/s] (**a**) and volume fraction (**b**) for different mesh sizes on the centerline of channel $\mathbf {A\cdot B}$.

The mesh independency analysis results performed for the microfluidic logic gates are shown in Fig. 4. From Fig. 4a, one observes that meshes 2 and 3 match very well for the viscoelastic phase volume fractions. Figure 4b shows the velocity magnitude for meshes 1, 2, and 3, and we verifiy a close match between mesh 2 and 3 also for the velocity field. As it displays the best balance between accuracy and computational cost, we concluded mesh 2 (107,888 nodes) is the most efficient and was used for all the following simulations performed in this paper.

#### Viscoelastic analysis

**Figure 5:**
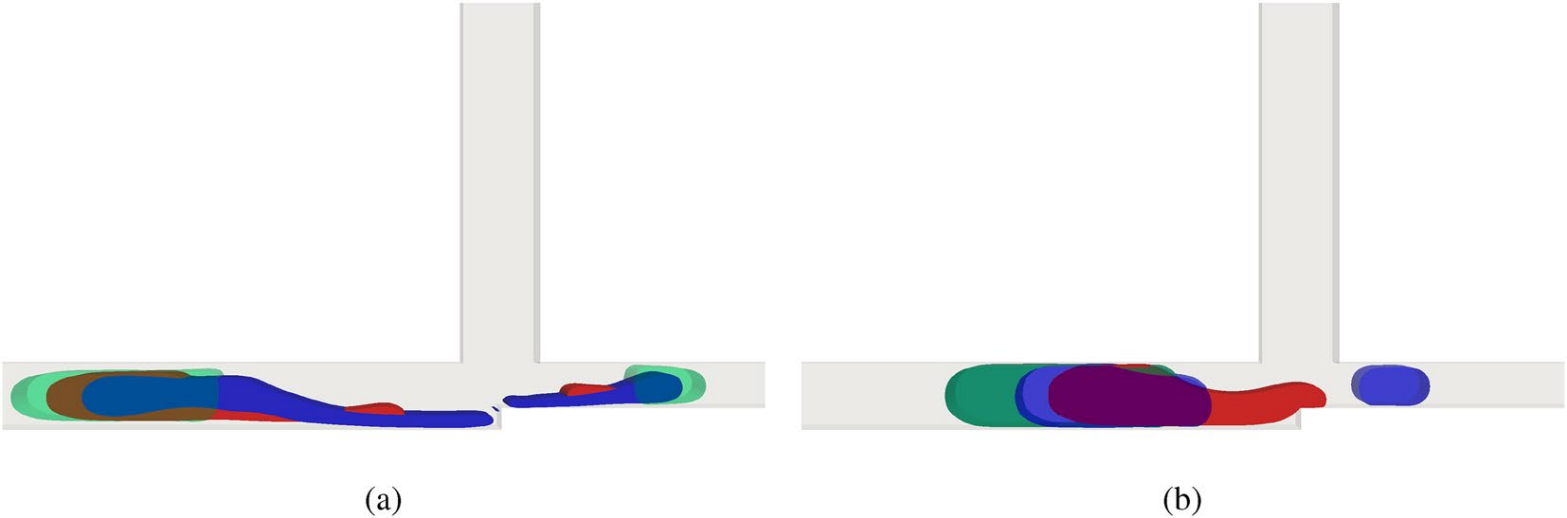
$\textbf{AND}$ logic gate for different fluid models with $Wi = 4$ and $L/H = 2.4$ for $Ca=0.1$ (**a**) and $Ca=0.01$ (**b**). The red fluid is the viscoelastic fluid, green is the Newtonian fluid and blue is the power-law fluid with $n=1.3$.

In this section, the importance of the viscoelastic effect is investigated. First, from Fig. 5a,b, one can see the difference between the three rheological models. The ratio of the droplet length and tube width is 2.4 and capillary number is 0.1 and 0.01 for Fig. 5a,b, respectively; The red fluid is a viscoelastic fluid with $Wi=4$. The blue fluid follows the power-law model, $\mu =K \left( \frac{1}{2}\sqrt{{\textbf{S}}:{\textbf{S}}} \right) ^{n-1}$ with $K=\mu _s$ and $n=1.3$; the green fluid is a Newtonian fluid with viscosity equal to $\mu = K = \mu _s$ with surface tension of 0.02 N/m, disregarding the contact angle effect. Figure 5a,b show the results in the cross-junction between tubes $\mathbf {A+B}$ and $\mathbf {A.B}$ for an $\textbf{AND}$ logic gate when the droplets should break. One can see from Fig. 5a that the rheological model strongly influences the breakage dynamics. When the capillary number is 0.1, the logic gate is always operational. One can also observe that for the non-Newtonian fluids (either for viscoelastic or power-law model) there is a long “tail” of the fluid before its breaks. This behavior is not present in the Newtonian fluid. Note that for the capillary number is 0.1, the surface tension forces acting across the interface are weaker than the case with the capillary number 0.01, which provokes droplet breakage. In Fig. 5b, it is observed that there is also a longer tail for non-Newtonian fluids, but the operational condition is only feasible for a power-law model. In Fig. 5b, the power-law fluid operates correctly even for a low capillary number. This occurs because the local capillary number increases due to local viscosity increases in regions with shear rate increases (splitting region).

For power-law model the operation regimes were studied by^8^. Their research highlighted a fascinating phenomenon: as one increases the droplet length, capillary number, and power-law index, the operating range of the AND state expands, while that of the OR state contracts. This qualitative trend becomes evident in our own investigation. However, our focus lies in discerning disparities between operational conditions across different models, and we have indeed identified scenarios where these models diverge. Since power-law models are solely dependent on shear rate, we recognize that the Oldrog-B model can outperform power-law models, especially in capturing viscoelastic effects, owing to its fundamental theoretical basis for this class of fluids.

**Figure 6:**
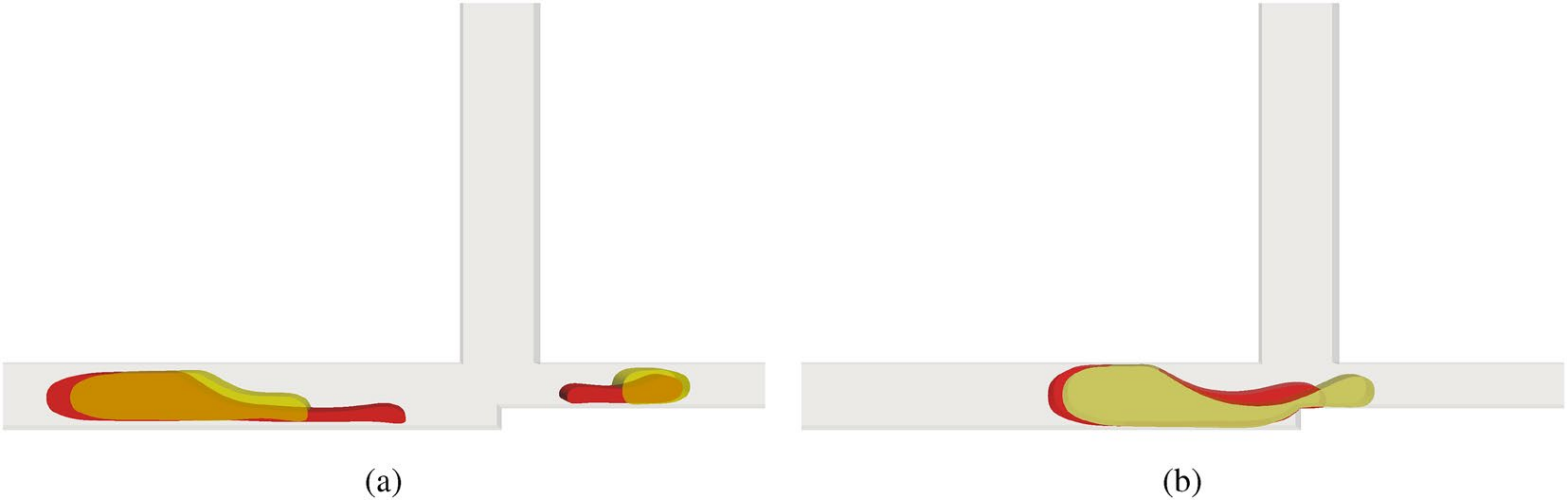
Influence of the Weissenberg number (Wi) for different capillary numbers of the viscoelastic fluid. The yellow fluid has $Wi = 0.01$ while the red fluid has $Wi = 4$. Both fluids have $Ca = 0.1$ (**a**) and $Ca = 0.01$ (**b**).

Figure 6a,b compare the influence of the Weissenberg number (Wi) for different capillary numbers. The yellow fluid has $Wi = 0.01$ while the red fluid has $Wi = 4$. We can see that for low Ca, the $\textbf{AND}$ logic gate becomes non-operational for both cases. Figure 6a shows that when the elastic forces are dominant, the fluid tends to deform and form a long tail. In Fig. 6b, when the viscous forces are dominant, a tail is formed, but it is very prolonged due to the reduction of the capillary numbers. In this case, Ca is 10 times smaller and viscous and elastic forces are not able to break the droplets.

**Figure 7:**
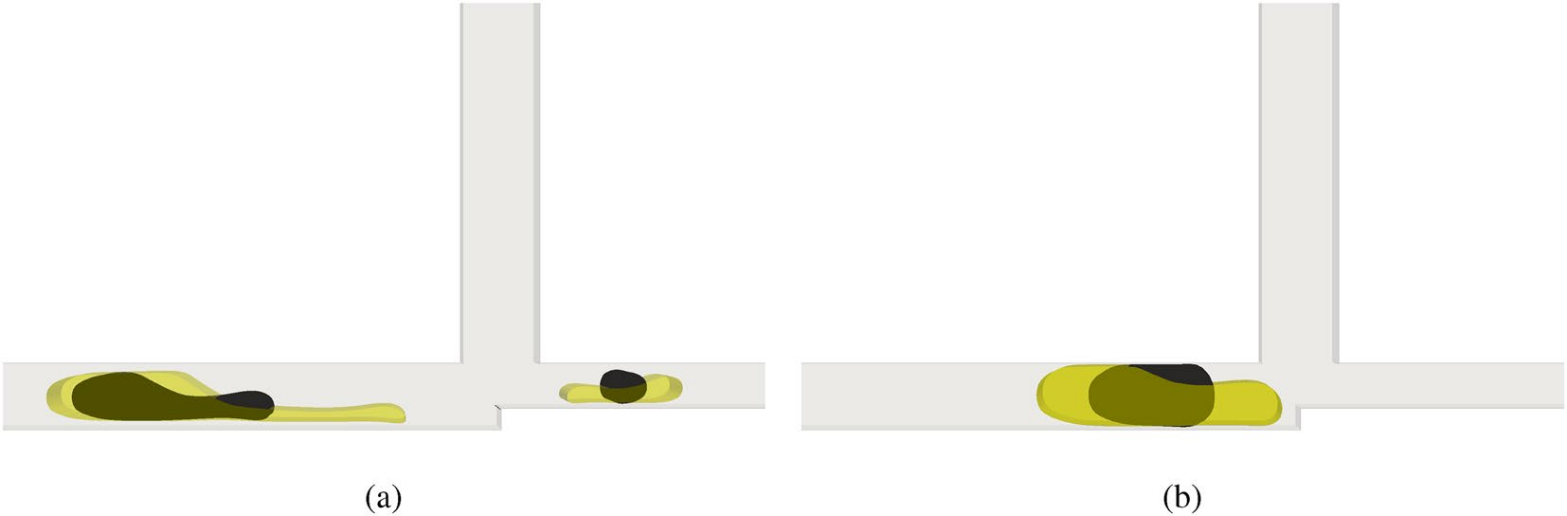
Comparison of the droplet initial size for the $\textbf{AND}$ logic gate for $Wi = 4$ for ratios *L*/*H* equal to 2.4 (yellow droplet) and 1.4 (black droplet) for $Ca =0.1$.

The initial size of the droplet is an important parameter in logic gates devices. Figure 7a,b compare the droplet initial size for the $\textbf{AND}$ logic gate for $Wi = 4$ for ratios *L*/*H* equal to 2.4 (yellow droplet) and 1.4 (black droplet). We can see that the size influences the droplet when it is crossing the cross-junction. As expected, larger droplets are more likely to split since they experience a larger deformation, while smaller droplets experience a lower deformation and hence are less likely to break.

**Figure 8:**
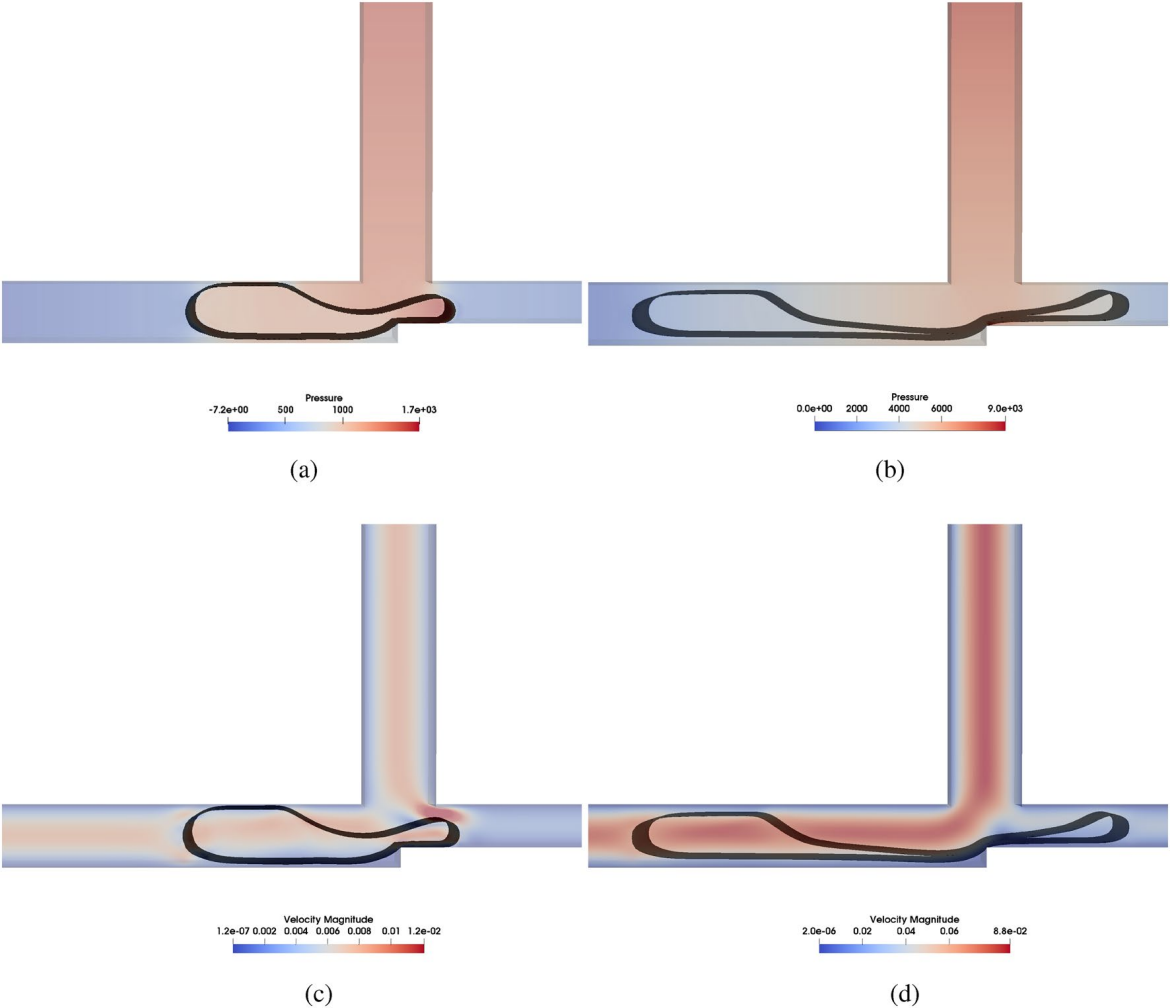
Comparison of the pressure field [Pa] (**a**,**b**) and the velocity magnitude fields [m/s] (**c**,**d**) for *AND* logic gate with $Wi =4$ and $L/H = 2.4$ with $Ca = 0.1$ (**a**,**c**) and $Ca = 0.01$ (**b**,**d**).

Figures 8 show the pressure and velocity profiles, respectively, for $Wi =4$ and $L/H = 2.4$ with $Ca = 0.01$ and $Ca = 0.1$ during the **AND** breaking (or not) process in the cross-junction. From Fig. 8a, we can see a considerably higher pressure in Tube ${\textbf{C}}$ for both cases. It was expected since the droplet blocks the connection between tubes $\mathbf {A+B}$ and $\mathbf {A.B}$. It is also expected that the pressure will increase as the convective term becomes dominant compared to the surface force if one increases the Ca. In Fig. 8b, we can observe the consequences of the higher pressure for $Ca = 0.1$; the fluid tends to break easily, as is expected since the energy to break the interface is less than for $Ca = 0.01$. This result plays an important role in the critical operational condition point. according to our result, the Ca is the most important parameter in the logic gate operational condition. This will be further described in the next section.

**Figure 9:**
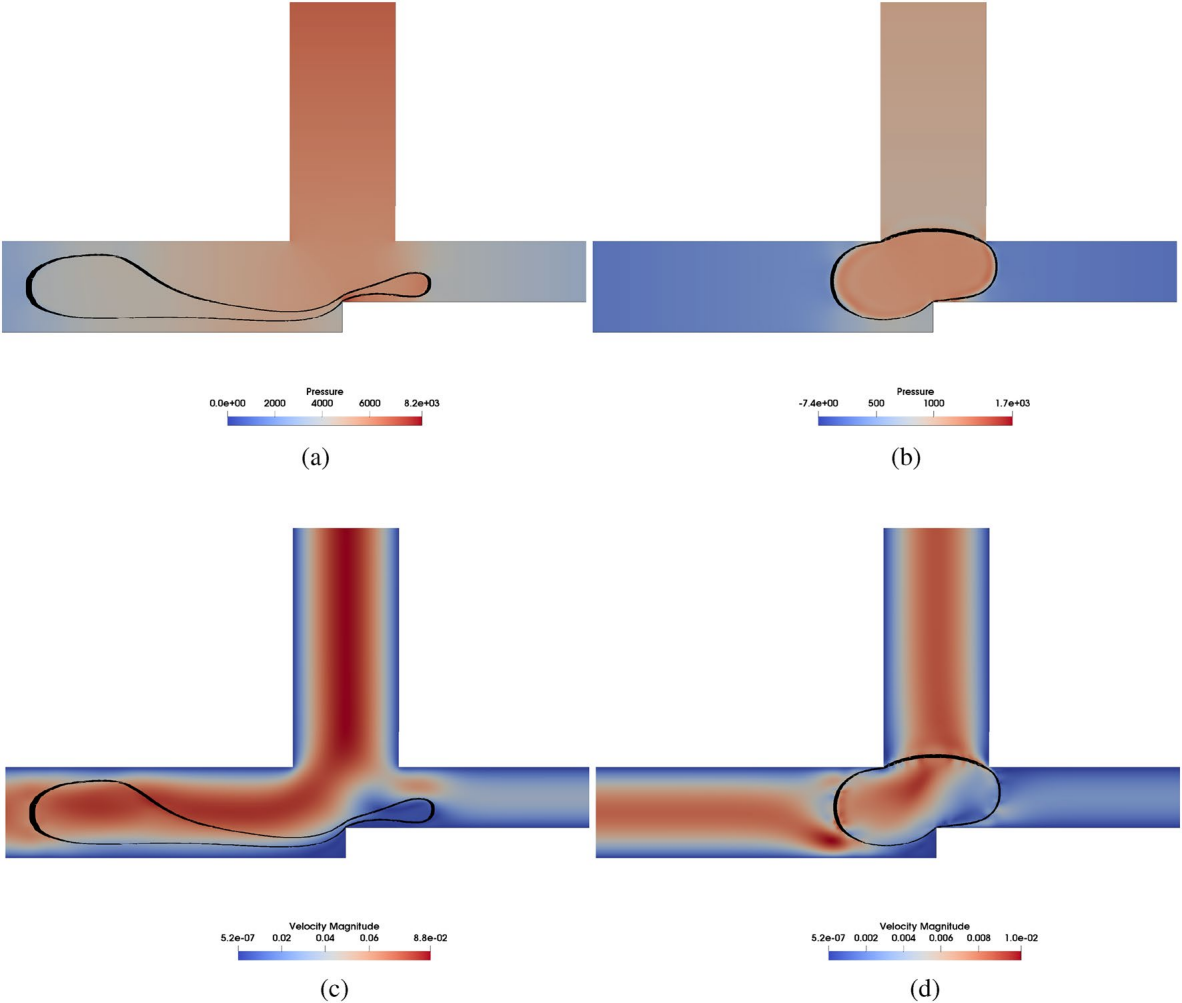
Pressure field [Pa] (**a**,**b**) and Velocity magnitude [m/s] (**c**,**d**) fields for *OR* logic gate with $Wi =4$ and $L/H = 2.4$ with $Ca = 0.1$ (**a**,**c**) and $Ca = 0.01$ (**b**,**d**).

Figure 9 show the pressure and velocity profiles, respectively, for $Wi =4$ and $L/H = 2.4$ with $Ca = 0.01$ and $Ca = 0.1$ during the **O** **R** breaking (or not) process in the cross-junction. Figure 9a,b are the pressure profiles, and the results show that if one increases *Ca* it is more likely to break the droplet. However, in this case, as it is a $\textbf{OR}$ logic gate, the breakage is not intended, and therefore, a low *Ca* number is required.

#### Electric field effect

An operational condition occurs when a predefined droplet logic gate is performed properly by the droplet, otherwise is a non-operational condition. In order to guarantee operational conditions, here we propose to control a droplet-based microfluidic device with an external electrical force. We include two electrodes at the whole walls of the tube $\mathbf {A.B}$ to create an additional force (produced by an electric potential difference) that attracts or repels a droplet in a 2D cross-junction. For the $\textbf{AND}$ logic gate, this external force should attract the droplet and force it to break in two. This task is not straightforward because the potential applied to the electrodes should be strong enough to attract the droplet but weak enough to not attract the whole droplet instead of breaking it. For the $\textbf{OR}$ logic gate, this external force should repel the droplet. In this last case, this is more straightforward since the force should only be strong enough to repel the droplet to avoid a non-operational condition. It is important to point out that electric fields is being used to manipulate droplets for different applications^2^, such as: elestrospray and electrocoalescence. The application of an electric field affects the surface energy of the droplet, enabling precise control over its movement and behavior. Moreover, this process induces a non-uniform charge density within the droplet (as described in Equation 12) that can be leveraged to exert control over the droplets. In this section, we proved that the logic gate regimes can be altered by the electric field.

**Figure 10:**
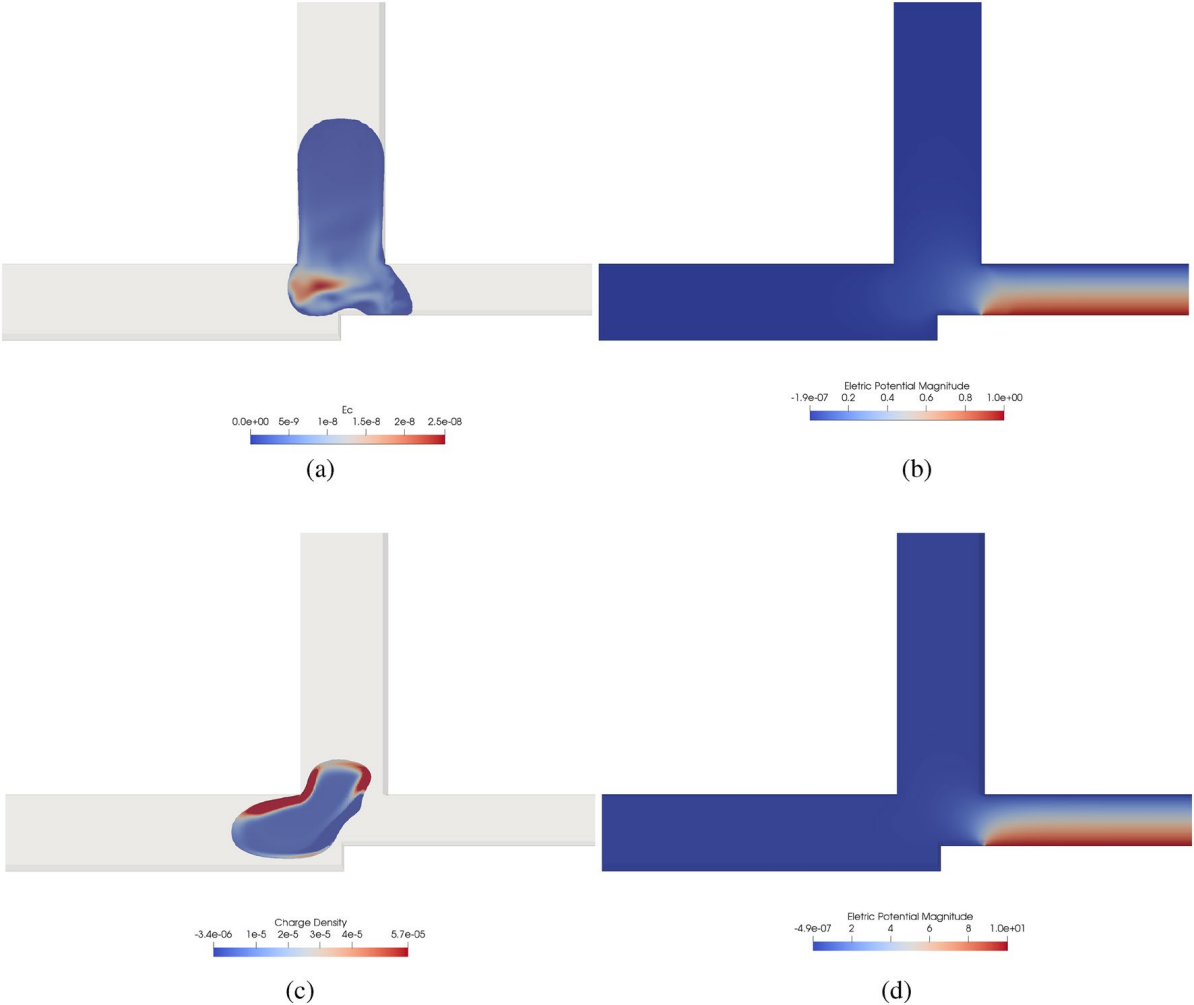
Electric charge distribution [C/m$^3$] (**a**,**c**) and the electric potential field [V] (**b**,**d**) for *AND* (**a**,**b**) and *OR* (**c**,**d**) logic gate for $Wi =4$ and $L/H = 2.4$ with $Ca = 0.01$ where the logic would be non-operational without an electric field.

**Figure 11:**
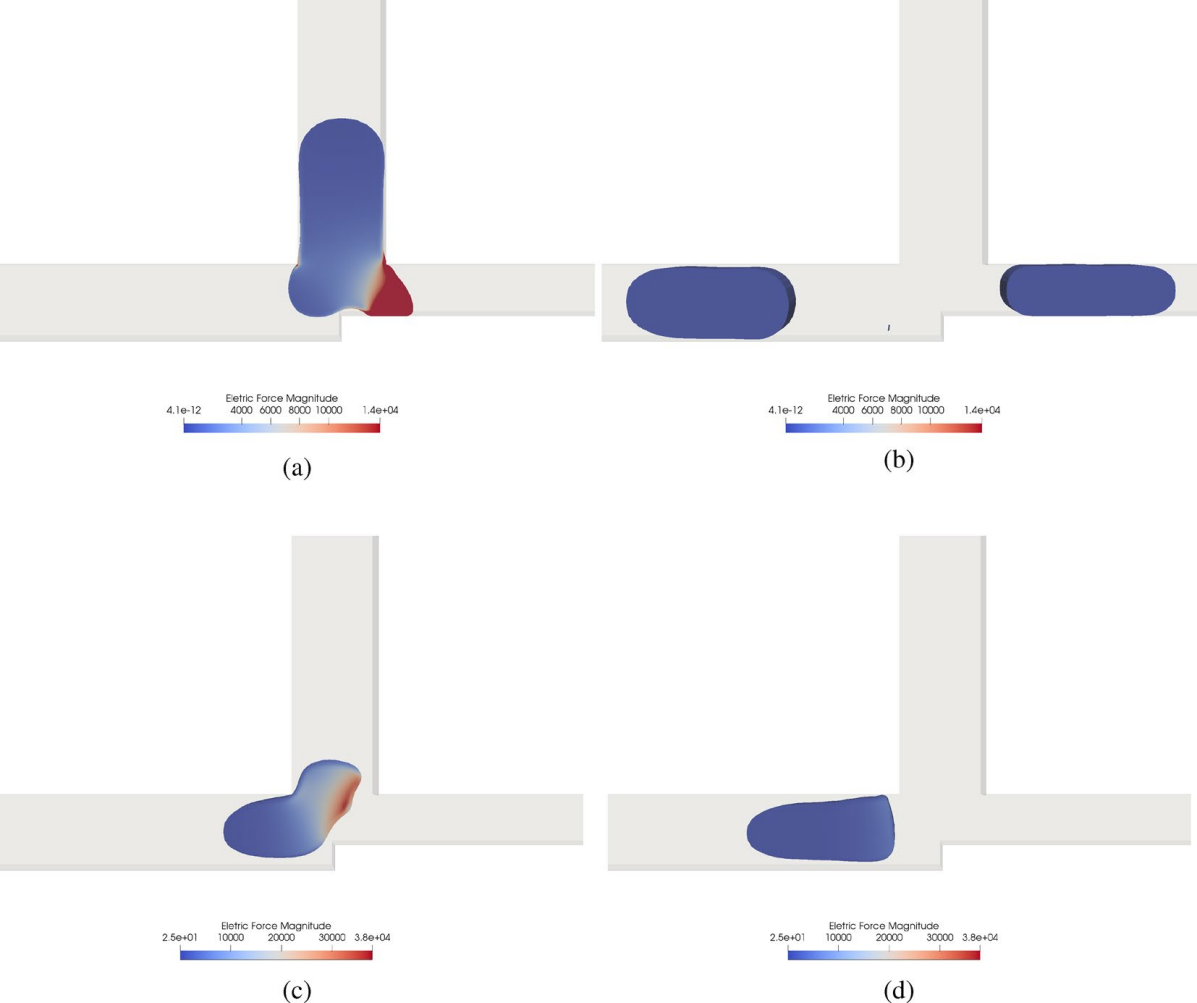
Electric force magnitude field [N/m$^3$]in *AND* (**a**,**b**) and *OR* (**c**,**d**) logic gates for $Wi =4$ and $L/H = 2.4$ with $Ca = 0.01$ where the logic would be non-operational without an electric field, before (**a**,**c**) and after (**b**,**d**) the droplet is pushed to $\mathbf {A+B}$.

Figure 10a,b show results for the $\textbf{AND}$ logic gate for $Wi=4$ and $L/H = 2.4$ with $Ca = 0.01$, such that the logic gate would be non-operational a priori. However, applying an electric force in a fluid with $R = 1\times 10^{1}$, $Q =1\times 10^{-1}$ and $Ca_E = 1\times 10^{-10}$, one can see that the droplet is attracted (see Fig. 11a to the electrodes. Figure 11a shows the result an instant before the droplet breaks, while Fig. 11b shows the droplet after it is already broken. Suppose a strong electric force is applied instead of completing the logic gate; in that case, the droplet is stuck in the tube $\mathbf {A.B}$. In these electrodes, the boundary conditions is a specified potential (based on the $Ca_E$) and with a zero initial charge concentration. In order to avoid trapping the droplet, the application of the external force application follows a step function with the frequency of half of the advective time. From Fig. 10a, one can see that the negative charge is concentrated near the electrodes with a positive potential (see Fig. 10b), thus attracting the droplet. This effect depends on the fluid properties; however, it is beyond the scope of the present manuscript to evaluate the fluid properties in the droplet attraction. We only aim to show that an external electric force can turn a non-operational system into an operational one.

In Fig. 11, we study an $\textbf{OR}$ logic gate for $Wi =4$ and $L/H = 2.4$ with $Ca = 0.01$, where the logic would be non-operational in the absence of an electric field. When we applied an electric force, with $R = 1\times 10^{-6}$, $Q =1\times 10^{5}$ and $Ca_E = 3\times 10^{-11}$, the droplet was repelled and the system became operational. However, in that case, there is no need to apply a periodic potential in the electrodes since the droplet can be stuck. Figure 11c shows the droplet being “pushed” to tube $\mathbf {A+B}$. As is expected, the force is more intense near the electrodes. When the droplet enters the tube $\mathbf {A+B}$, the electric force effect reduces drastically, as can be seen in Fig. 11d. The negative charge is closer to the electrodes, and the electric force repels the droplet, as can be seen in Figs. 10c and 11c due to the electric field effect, see Fig. 10d.

### Binary classification ROM

In binary logic gates, the logic is classified into two states. Instead of creating a complex regime map separated by logic and parameters as in^8^, we modelled the binary logic with a reduced-order model (ROM) based on a binary deep neural network. Thus, a general formulation can be based on critical dimensionless parameters. In order to produce data for the training, several simulations were performed for $\textbf{AND}$ and $\textbf{OR}$ logic gates, see Table 1. The data set has 210 entries for each logic gate, with a total of 420 samples.

**Table 1:** Dimensionless parameters used in the training step (total of 420 data).

Dimensionless parameter	Cases
$Ca = \frac{\mu U}{\gamma }$	$1\times 10^{-3}$, $2\times 10^{-3}$, $5\times 10^{-3}$, $1\times 10^{-2}$, $2\times 10^{-2}$, $5\times 10^{-2}$, $1\times 10^{-1}$
$Wi = \frac{U \lambda }{H}$	$1\times 10^{-2}$, $5\times 10^{-2}$, 1.0, 2.0, 4.0
$\frac{L}{H}$	1.4, 1.6, 1.8, 2.0, 2.2, 2.4

TensorFlow was used for the classification model training. We employ the binary cross-entropy loss function and Adam optimization algorithm optimizer for binary classification. The binary cross-entropy is very robust in solving non-linear multidimensional classification problems^50^. Figure 13 shows the deep neural network accuracy for the training and validation step for a simple topology with 10 neurons with 3 layers. For the first two layers, relu and for the last, sigmoid activation functions were used. For the model generation, we used a total of 3000 epochs with a batch size of 500, considering 20% for validation and 10% for testing our classification model. Finally, the accuracy in the test (unseen data) evaluation was 92%. If AND or OR logic gates work, the classification model would return 1; otherwise, 0 without needing a regime map. This result can be used to estimate the viability of a logic gate without expensive simulations. The average computational time for each simulation is around 2 h in a computer with an Intel Xeon E5-2640 v4 2.4 GHz but using the classification model takes less the 1 s.

**Figure 12:**
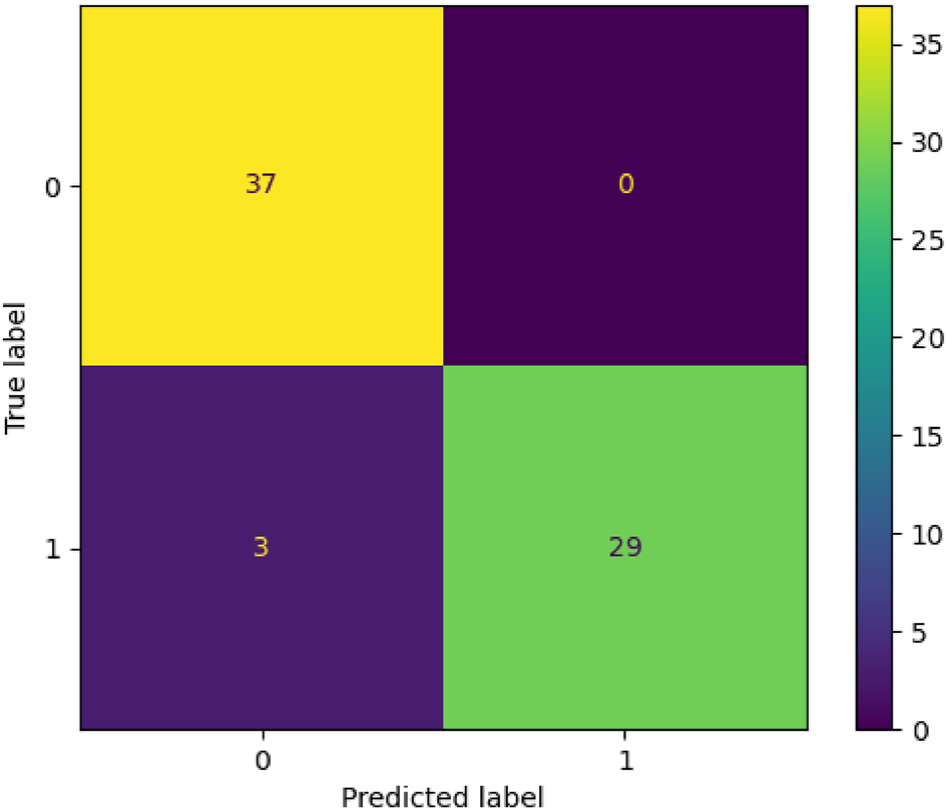
Confusion matrix over a test set.

In Fig. 12, we analyze how well a classification model performs. The confusion matrix provides a snapshot of the model’s predictions versus the actual outcomes, allowing us to calculate different performance metrics based on false and true responses. This classification model excels in prediction accuracy, as shown by the dominant diagonal in the confusion matrix.

**Figure 13:**
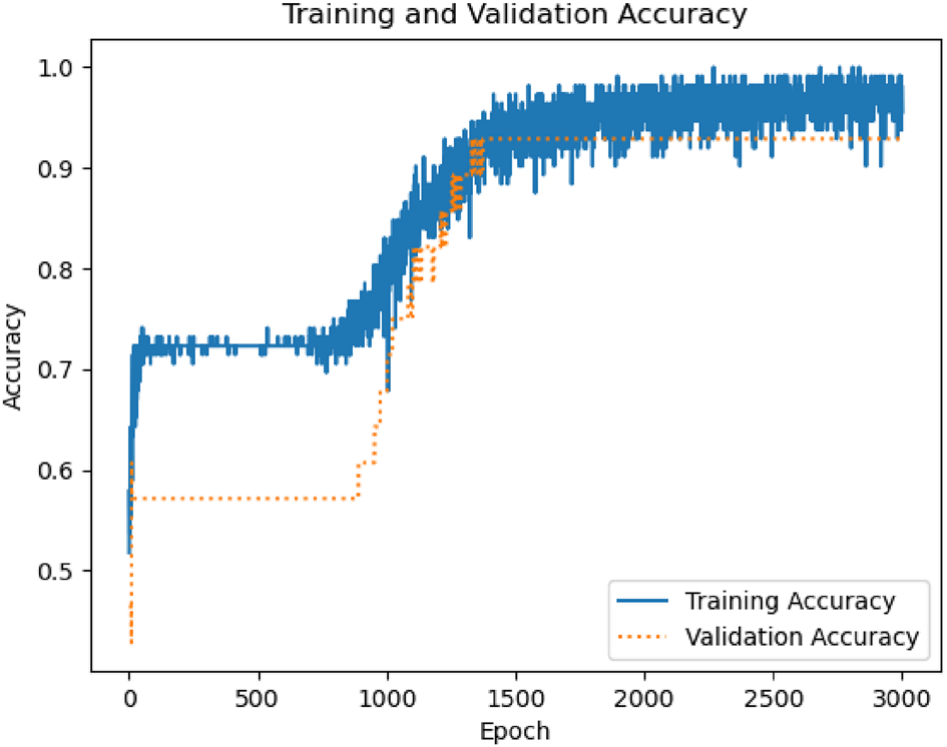
Classification accuracy for training and validation.

## Conclusions

In our study on droplet-based logic gate systems operating with viscoelastic fluids, we investigated the influence of viscoelasticity on the operational conditions using the AND/OR system proposed by^48^ as a basis. We observed that the behavior of viscoelastic fluids, following the power-law model, can significantly impact the dynamics of the logic gates compared to Newtonian fluids. Therefore, it is crucial to consider the rheological model to obtain realistic results, and simplifications using either a Newtonian or power-law model may lead to unrealistic conclusions. To understand the system dynamics, we studied the Weissenberg number (Wi), the Capillary number (Ca), and the geometric droplet parameters. We found that all these parameters can influence the system dynamics, but the Capillary number has emerged as the most important factor, which is consistent with the findings reported by^8^. To improve the prediction of operational conditions and accelerate the process without relying on complex simulations, we employed a deep learning (DL) classification algorithm to develop a reduced-order model. This DL model enabled us to predict the operational conditions beyond the range of our existing data. Furthermore, we demonstrated that non-operating regions can become operational by applying an external force. Although we did not specifically analyze the influence of fluid properties in this study, we showed that applying an electric field to the outlet tubes using an electrically induced technique can transform the system from non-operational to operational conditions. Overall, our research highlights the importance of considering viscoelastic properties and the rheological model in droplet-based logic gate systems. The developed DL model provides a valuable tool for predicting operational conditions and offers insights into the potential for external interventions to induce system operability. Future studies may explore the influence of fluid properties and further optimize the electrically induced technique to enhance the performance and applicability of these systems.

## Data Availability

The data generated and analyzed in this study is available from the corresponding author on reasonable request.
